# Characterization and applications of iron oxide nanoparticles synthesized from *Phyllanthus emblica* fruit extract

**DOI:** 10.1371/journal.pone.0310728

**Published:** 2024-09-19

**Authors:** Easha Fatima, Iqra Arooj, Hamna Shahid, Abida Aziz

**Affiliations:** 1 Department of Microbiology & Molecular Genetics, Faculty of Life Sciences, The Women University, Multan, Pakistan; 2 Department of Botany, Faculty of Life Sciences, The Women University, Multan, Pakistan; Chaudhary Devi Lal University, INDIA

## Abstract

Nanotechnology is a treasure trove of diversified themes which are endowed with broad applications. Herein, iron oxide (Fe_2_O_3_) nanoparticles were synthesized using *Phyllanthus emblica* aqueous fruit extract. The UV-Visible spectrum exhibited a surface plasmon resonance peak at 295nm. Fourier Transform Infrared Spectroscopy provided insight into the functional groups responsible for capping. X-ray diffraction analysis authenticated the crystalline nature of nanoparticles, while energy dispersive X-ray spectroscopy divulged that iron and oxygen comprised 54% of the nanoparticles’ weight. Scanning electron microscopy established irregular morphology and agglomeration of nanoparticles. The Fe_2_O_3_ nanoparticles validated potent antimicrobial activity against 11 bacterial and 1 fungal isolates. The biggest zone of inhibition (23mm) was measured against *S*. *enterica*, whereas the smallest zone of inhibition (12mm) was documented against *C*. *albicans* and *E*. *coli*. The values for minimum inhibitory concentration ranged between 10 and 15μg/ml for all microbes. Nevertheless, no synergy was exhibited by the nanoparticles with any of the selected antibiotics (Fractional Inhibitory Concentration Index > 1). The photocatalytic dye degradation capability of Fe_2_O_3_ nanoparticles was assessed and the observations implied a significant increase in degradation of methyl red although, not of methylene blue. Furthermore, the nanoparticles were in possession of substantial antioxidant (34–38%) and anti-inflammatory (31–38%) capacities. Consequent upon the robust activities of *P*. *emblica*-mediated nanoparticles, these can be scrutinized for biomedical and environmental implementations in future.

## Introduction

Nanotechnology, the engineering of functional systems at the molecular scale, stands at the forefront of scientific innovation, bridging multiple disciplines including chemistry, physics, and biology [1]. It is the science of nanomaterials having size ranging from 1 to 100nm [2]. Among its diverse applications, the synthesis of nanoparticles, including metal oxide nanoparticles, has garnered significant attention due to their potential uses in biomedicine, magnetic storage, and environmental remediation [3, 4].

Iron oxide (Fe_2_O_3_) nanoparticles are particularly valued for their magnetic properties, biocompatibility, and environmental stability [5]. They have been widely researched for diversified applications including, for instance, targeted drug delivery, MRI contrast enhancement, in addition to their use as agents for hyperthermia treatment of cancer [6]. Fe_2_O_3_ nanoparticles are of various types but their most popular and promising candidates comprise maghemite, magnetite, and hematite as their biocompatibility has been confirmed [7, 8].

The conventional synthesis of Fe_2_O_3_ nanoparticles often involves physical or chemical methods, which, while effective, may utilize hazardous chemicals and energy-intensive processes that are not environment-friendly [9]. In response to these challenges, green synthesis methods have emerged, utilizing biological entities such as plant extracts that function as stabilizing as well as reducing agents during synthesis [10–12]. Some latest findings have documented the synthesis of Fe_2_O_3_ nanoparticles from leaf extracts of *Carica papaya*, *Laurus nobilis* and *Psidium guajava* [13–15].

Among the various botanicals explored, *Phyllanthus emblica*, known by the common name of Indian gooseberry, has shown promise due to its rich content of ascorbic acid, tannins, and flavonoids [16, 17]. *P*. *emblica* plant extracts have been employed in the synthesis of various metallic nanoparticles including magnesium oxide, iron oxide, gold, silver, copper, and zinc sulfide nanoparticles, for instance [16, 18–22]. The phytochemical constituents of *P*. *emblica* extracts act as natural antioxidants and can effectively mediate the formation of Fe_2_O_3_ nanoparticles, offering a pathway to more sustainable nanoparticle synthesis [23, 24]. Studies have demonstrated that the *Phyllanthus emblica* fruit extract can reduce iron salts to form iron oxide nanoparticles with controlled sizes and shapes [21]. This bio-inspired approach not only presents a low-cost and eco-friendly alternative but also aligns with the principles of green chemistry, reducing the environmental footprint of nanoparticle production [10, 25].

From what we know, Fe_2_O_3_ nanoparticles have been synthesized by utilizing *P*. *emblica* plant extract, but their antimicrobial potential has not been explored. So, current experimental study focuses on synthesis of Fe_2_O_3_ nanoparticles from *P*. *emblica* fruit extract, their characterization using several analytical methods and the investigation of their antimicrobial, anti-inflammatory, antioxidant, and photocatalytic dye degradation potentials. Our findings suggest that the nanoparticles synthesized using this method may offer an easy, rapid, cost-effective, and environment-friendly alternative to currently employed therapeutic agents like antibiotics. Additionally, research is ongoing to find the exact mechanisms by which plant phytochemicals influence nanoparticle formation, with the aim of refining the synthesis process for industrial applications.

## Materials and methods

### Preparation of *Phyllanthus emblica* fruit extract

Dried *P*. *emblica* fruits, natively known as Amla, were obtained from a local market of Multan city. Fruits were cleaned to remove the impurities and grinded into a fine consistency. *P*. *emblica* fruit extract was formulated in three distinct concentrations ie, 2%, 4%, and 8%, by dissolving 2g, 4g and 8g of the dried fruit powder in 100 ml distilled water, respectively. These mixtures were then heated to 80°C for 30 minutes, while being stirred continuously. After heating, the extract was cooled to ambient temperature (28°C), passed through a Whatmann filter paper no. 01, and immediately utilized for the production of Fe_2_O_3_ nanoparticles.

### *P*. *emblica*-mediated synthesis of Fe_2_O_3_ nanoparticles

**Fig 1:**
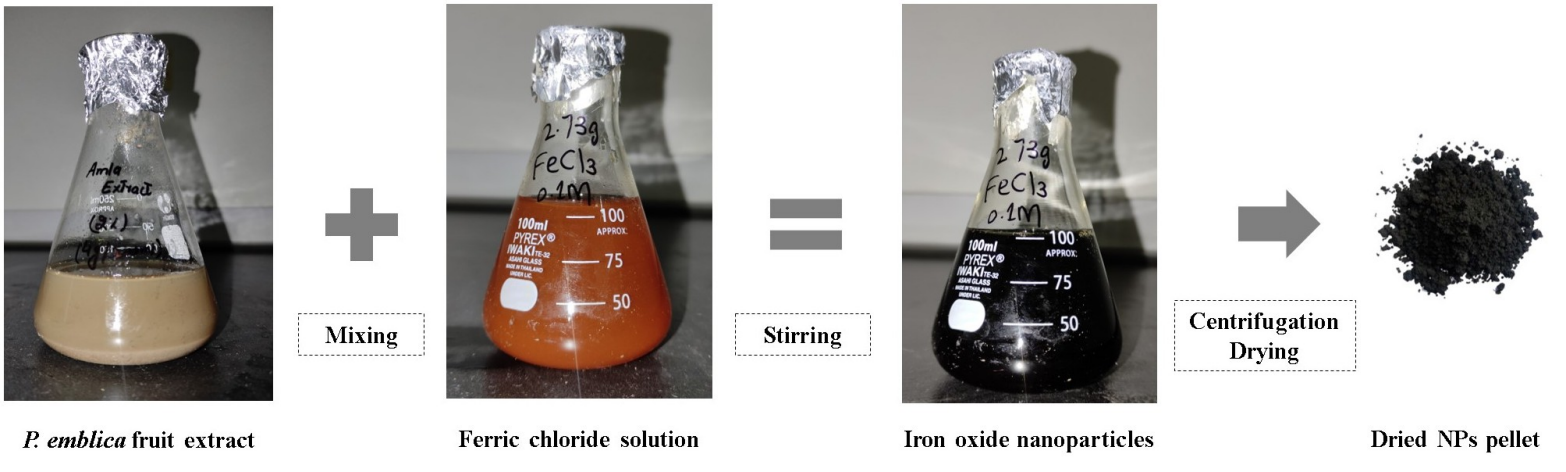
The production of Fe_2_O_3_ nanoparticles from *P*. *emblica* fruit extract.

For the preparation of Fe_2_O_3_ nanoparticles, ferric chloride was used as the metal source while *P*. *emblica* fruit extract served as the reducing and capping agent (Fig 1). Fe_2_O_3_ nanoparticles were produced using different concentrations of fruit extract ie, 2%, 4% and 8%. 50 ml of the freshly prepared nanoparticle solution was slowly added to 50 ml of 0.1M ferric chloride solution in 1:1 ratio [26]. 1M sodium hydroxide was used to adjust the pH of this solution to 11. An instant color change was noticed. Nanoparticle solution was stirred for 30 minutes to get an intense black color, which indicated the synthesis of Fe_2_O_3_ nanoparticles. The solution was centrifuged at 8000 r.p.m. for 20 minutes with subsequent washing of pellet using distilled water. The pellet was dried using hot air oven and converted to powder by grinding. All the variations of Fe_2_O_3_ nanoparticles were tested for their antimicrobial potential and the one with the greatest activity was utilized for further testing.

### Characterization of Fe_2_O_3_ nanoparticles

To determine the physicochemical properties of Fe_2_O_3_ nanoparticles, various techniques were employed. UV-Vis spectroscopy was performed at 200–600 nm for confirming the formation of nanoparticles. Moreover, Fourier transform infrared (FTIR) spectroscopy was done at 500–4000 cm^-1^ to study the chemical properties of Fe_2_O_3_ nanoparticles. X-ray diffraction (XRD) was performed to reveal the crystalline structure of the nanoparticles utilizing a 50–500μm X-ray beam. Scanning electron microscopy (SEM) was performed for observing physical as well as mechanical properties of Fe_2_O_3_ nanoparticles at a magnification up to 1000X. Energy dispersive X-ray (EDX) investigation was accomplished to evaluate the occurrence of certain elements in the nanoparticle solution at 20kV and 270x.

### Antimicrobial activity of Fe_2_O_3_ nanoparticles

To determine the antimicrobial activity of Fe_2_O_3_ nanoparticles, agar well-diffusion method was employed. A range of pathogenic microbes were isolated in the Pathology Department of Nishter Hospital Multan, in November 2022. A total of 11 clinically isolated bacteria (including *Staphylococcus aureus*, *Pseudomonas aeruginosa*, *Proteus vulgaris*, *Klebsiella pneumoniae*, *Escherichia coli*, *Enterobacter aerogenes*, *Salmonella enterica*, *Acinetobacter baumannii*) and 1 fungus (*Candida albicans*) were included in this study. Mueller-Hinton agar (MHA) was prepared and poured in sterilized petri plates. After standardizing all the test microorganisms by using 0.5 McFarland standard, they were applied onto MHA plates by using sterile cotton swabs. Plates were allowed to dry. Subsequently, a sterile Pasteur pipette was used to make three wells on every petri dish. 50 μl Fe_2_O_3_ nanoparticles were added to every well and allowed to infuse in the surrounding agar. Plates were incubated at 37°C for 24 hours. Following incubation, the zones of inhibition were measured in millimeters and noted.

### Minimum inhibitory concentration

The lowest concentration at which Fe_2_O_3_ nanoparticles inhibited microbial growth, known as the minimal inhibitory concentration (MIC), was ascertained using a recognized broth dilution technique. The test organisms were cultivated in a nutrient-rich medium overnight and then adjusted to standard concentration before being utilized. 100 μl of prepared nutrient broth was filled in the wells of a sterile 96-well plate. Starting with 50 μg/ml of Fe_2_O_3_ nanoparticles in the 1^st^ well, serial two-fold dilutions were prepared, ending with a concentration of 0.1 μg/ml in the 10^th^ well. To each well, 50 μl of the standard growth mixture was added. 11^th^ and 12^th^ wells were correspondingly designated positive and negative controls. Incubation was done at 37°C overnight. MIC was determined both by direct observation and with the assistance of a microtiter plate reader, noting absorbance readings at 620 nm.

### Antibiotic sensitivity testing

For the antibiotic sensitivity analysis of the bacterial samples, the Kirby-Bauer disc diffusion approach was employed. Nine different antibiotics were chosen for this purpose comprising Clindamycin (CL-10), Imipenem (IPM-10), Ciprofloxacin (CIP-5), Gentamicin (CN-10), Ceftriaxone (CRO-30), Cefepime (FEP-30), Sulbactam (SCF-105), Trimethoprim/Sulfamethoxazole (SXT-25), and Ampicillin (AMP-10). MHA plates were set up and cultured samples were smeared on these by using sterile cotton swabs. Once these plates were dried, antibiotic discs from various categories were suitably placed on each plate. The incubation was done at 37°C overnight. Post the incubation, zones indicating bacterial sensitivity or resistance were measured. The findings were then documented in alignment with the standards set by the CLSI for 2023.

### Synergistic activity of Fe_2_O_3_ nanoparticles with selected antibiotics

To check the synergism between Fe_2_O_3_ nanoparticles and selected antibiotics ie, CRO-30, AMP-10, IPM-10 and CN-10, MHA plates were prepared. Bacterial samples were applied on them by using clean cotton swabs. Antibiotic discs, previously immersed in the nanoparticle solution, were then carefully positioned onto these plates in a sterile manner. The plates were incubated at 37°C for a span of 24 hours. Afterwards, the zone diameters were measured and noted. For evaluation of the combined efficacy, following formula was used to calculate the fractional inhibitory concentration index (FICI) values.


$$FICI=\frac{Antibacterial\;effect\;of\;NPs+Antibacterial\;effect\;of\;antbiotic}{Antibacterial\;effect\;of\;both\;NPs\;\&\;antibiotic}$$

### Photocatalytic dye degradation potential of Fe_2_O_3_ nanoparticles

The potential of Fe_2_O_3_ nanoparticles for photocatalytic dye degradation was established by the protocol of Dulta et al [27]. Methyl red as well as methylene blue solutions were prepared (20 ppm) in separate flasks followed by the measurement of initial dye concentration. 10 mg/L of Fe_2_O_3_ nanoparticles were added in these solutions, mixed for 15 minutes in dark and placed in the sunlight outside. At constant intervals, solutions were stirred. After 130 minutes, solutions were centrifuged to remove nanoparticles and final dye concentration was measured by spectrophotometry, at 525 nm and 625 nm. Percentage dye degradation was calculated using the formula:

$$\eta =\frac{C₀-Ct}{C₀}\;\times 100$$

(where, C₀ is initial dye conc. and C_t_ is final dye conc. after exposure).

### Antioxidant potential of Fe_2_O_3_ nanoparticles

Total antioxidant capacity (TAC) of Fe_2_O_3_ nanoparticles was ascertained employing the phosphomolybdenum technique. 28 mM sodium phosphate, 4 mM ammonium molybdate, as well as 0.6 M sulfuric acid were mixed in equal amounts to make working solution. Nanoparticle solutions of varying strengths, spanning from 200–800 μg/ml, were made in d.H_2_O. 1 ml of reagent mixture and 100 μl of different nanoparticle solutions were taken in separate test tubes, ensuring thorough mixing. Ascorbic acid was taken as standard. These tubes were then subjected to water bath set to 95°C for a duration of 90 minutes. If antioxidants are present, a greenish-blue hue emerges, denoting that phosphomolybdate ion has been reduced and phosphomolybdenum (V) complex has been formed. Post incubation, the tubes were allowed to cool to ambient temperature and spectrophotometric measurement of every specimen was recorded at 695 nm. TAC was then deduced with the help of equation given below.


$$TAC\;\%=\frac{\left(ODcontrol\;-\;ODsample\right)}{ODcontrol}\;x\;100$$

The experiment was repeated thrice and the mean was computed.

### Anti-inflammatory potential of Fe_2_O_3_ nanoparticles

To analyze the anti-inflammatory properties of Fe_2_O_3_ nanoparticles, a 0.2% BSA solution was used. Different nanoparticle concentrations (200 to 800 μg/ml) were synthesized followed by the addition of 5 ml BSA solution to each of them, separately. Incubation was done in water bath at 75°C for 5 minutes subsequent to which, they were brought to room temperature. Ascorbic acid was taken as control. For each sample, absorbance was measured at 660 nm. The relative anti-inflammatory efficacy was deduced as follows:

$$Anti-inflammatory\;activity\;\%=\frac{\left(ODcontrol\;-\;ODsample\right)}{ODcontrol}\;x\;100$$

The test was performed in triplicates and average value was reported.

## Results

### Visual analysis and UV-Vis spectroscopy of nanoparticles

**Fig 2:**
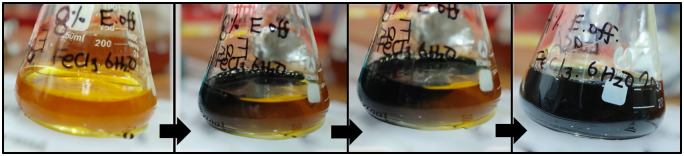
Color change from yellow to intense black provides visual indication of nanoparticle formation.

**Fig 3:**
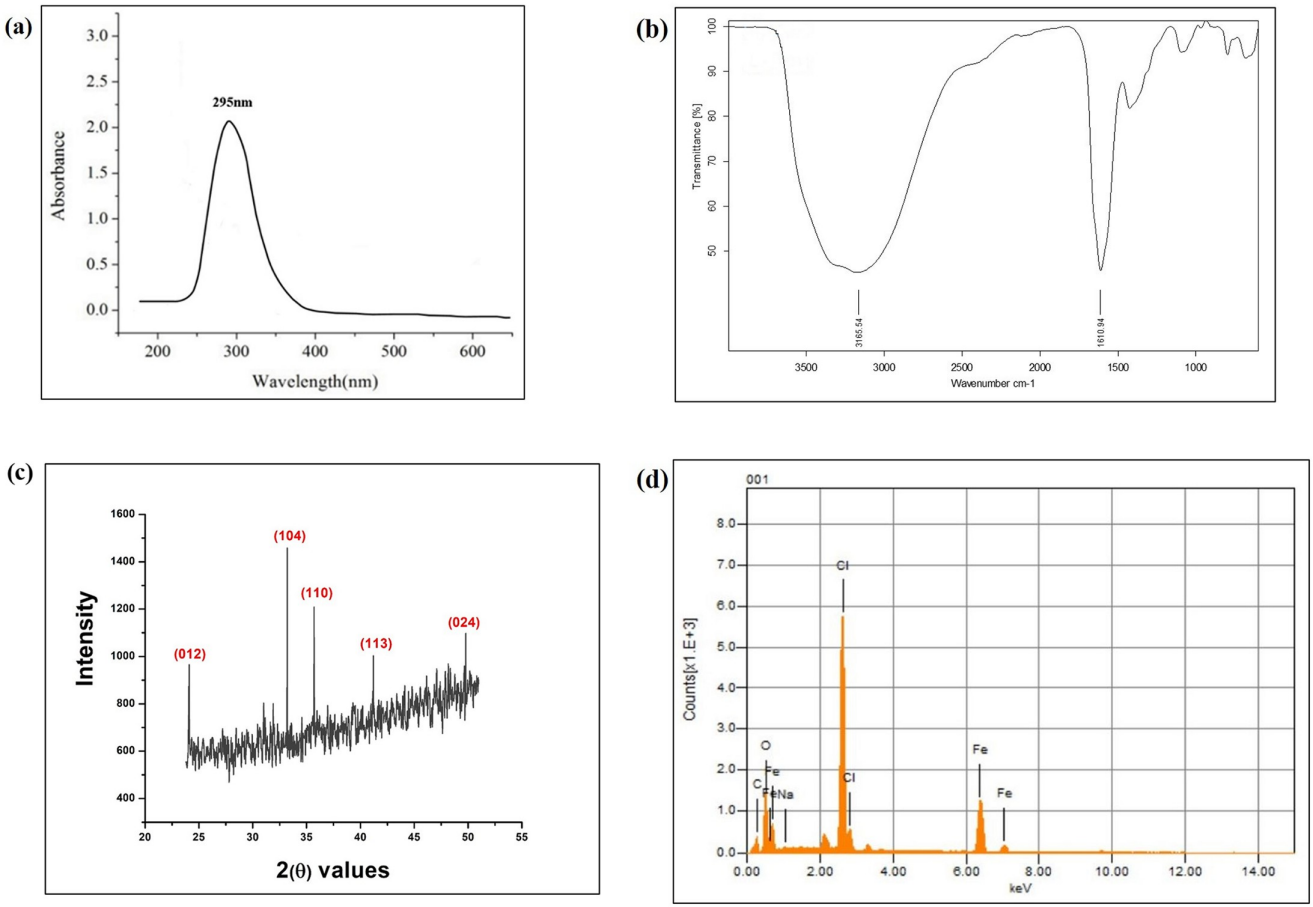
Characterization of nanoparticles. (a) UV-Vis spectrophotometry graph of Fe_2_O_3_ nanoparticles showing peak at 295–300 nm. (b) FTIR spectrum of Fe_2_O_3_ nanoparticles showing peaks at 3165 cm^−1^ and 1610 cm^−1^ (c) XRD graph showing multiple peaks. (d) EDX graph showing clear Fe and O peaks.

*P*. *emblica* fruit extract proved to be a nice source for synthesizing Fe_2_O_3_ nanoparticles. Upon mixing ferric chloride solution with this extract, an intense black color appeared readily which confirmed the formation of Fe_2_O_3_ nanoparticles (Fig 2). For further validation of nanoparticle synthesis, UV-Vis spectrum was obtained which presented a sharp peak at 295 nm (Fig 3a). Appearance of peak within the aforementioned range confirmed nanoparticle synthesis.

### Characterization of Fe_2_O_3_ nanoparticles

**Fig 4:**
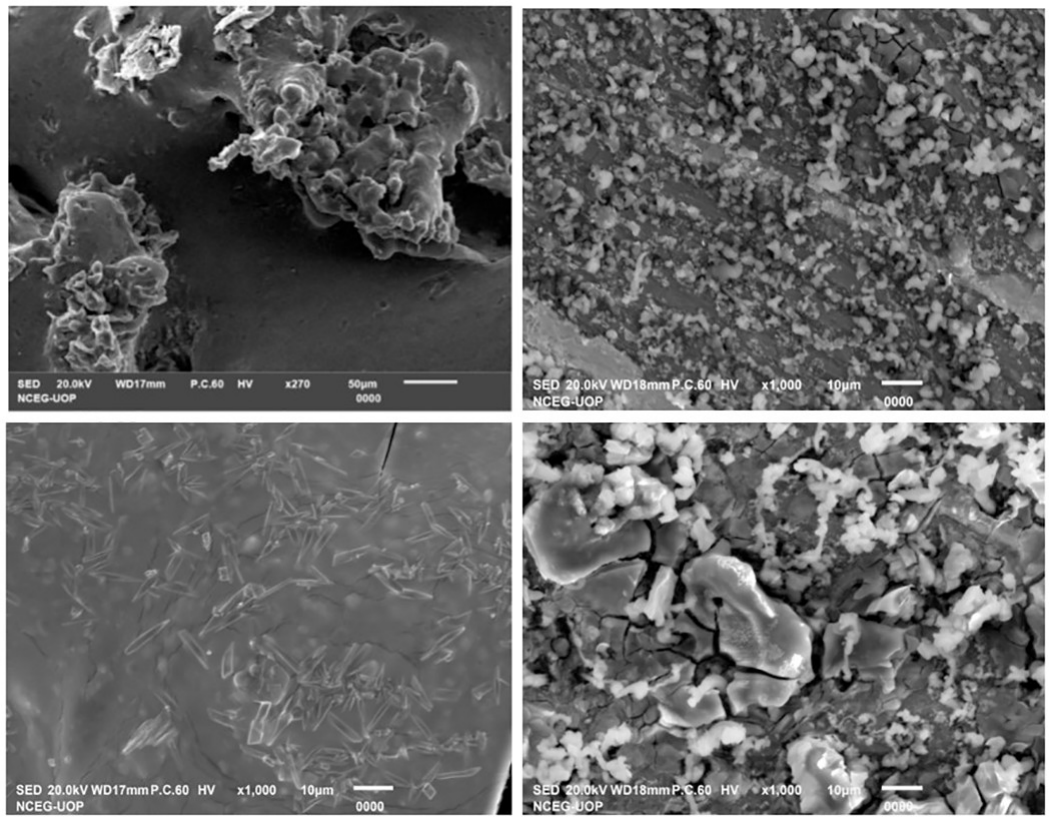
SEM images of Fe_2_O_3_ nanoparticles at different magnifications.

FTIR analysis of Fe_2_O_3_ nanoparticles presented peaks corresponding to the O-H bond (3165 cm^−1^) and the C = C bond (1610 cm^−1^) (Fig 3b). Peaks present in the region of 600 cm^−1^ signaled the occurrence of the Fe-O bond validating the formation of Fe_2_O_3_ nanoparticles. The presence of multiple FTIR peaks pointed towards various bioactive phytochemicals which might have adsorbed on the surface of nanoparticles during their green synthesis. XRD spectrum of Fe_2_O_3_ nanoparticles displayed peaks at 2 (θ) values of 24.1, 33.2, 35.7, 41.2, and 49.78, corresponding to the miller index planes of (012), (104), (110), (113) and (024) (Fig 3c). EDX analysis showed clearly visible peaks indicative of the occurrence of different elements in the nanoparticle sample (Fig 3d). EDX analysis presented that Fe and O were the most abundant elements in the sample cumulatively constituting 54% of the total weight and their peaks were located between 0.0 and 0.7 keV. Images acquired through SEM disclosed that the nanoparticles were irregular in shape and formed huge aggregates (Fig 4).

### Antimicrobial activity of Fe_2_O_3_ nanoparticles

**Fig 5:**
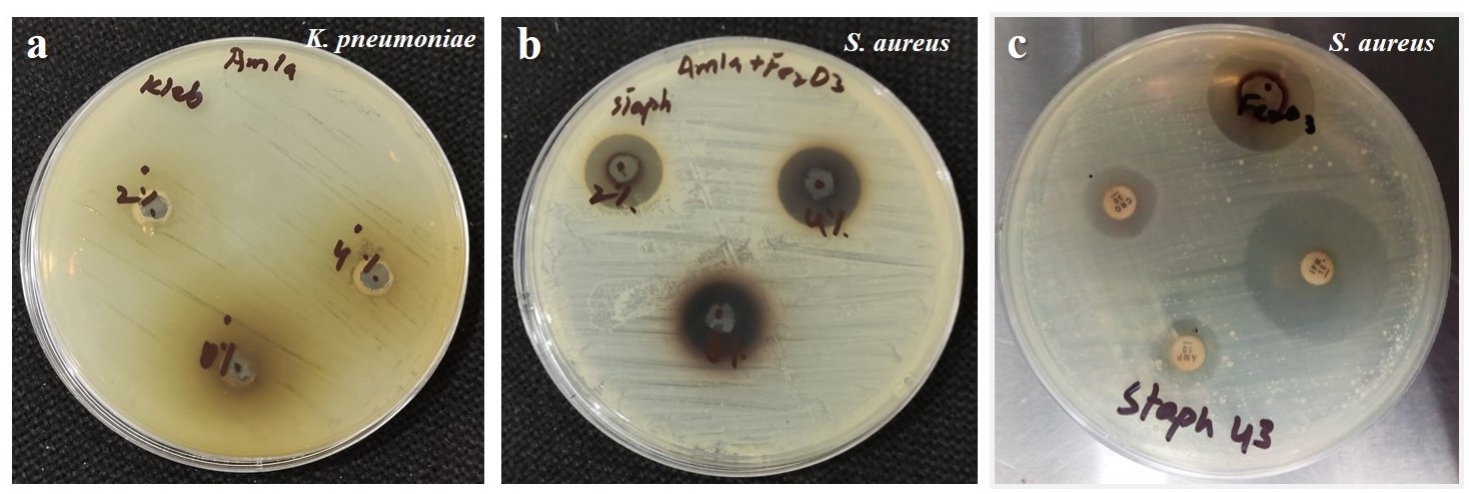
MH agar plates depicting (a) antibacterial effect of *P*. *emblica* fruit extract, (b) antibacterial activity of Fe_2_O_3_ nanoparticles (c) combined antibacterial effect of Fe_2_O_3_ NPs and selected antibiotics.

**Fig 6:**
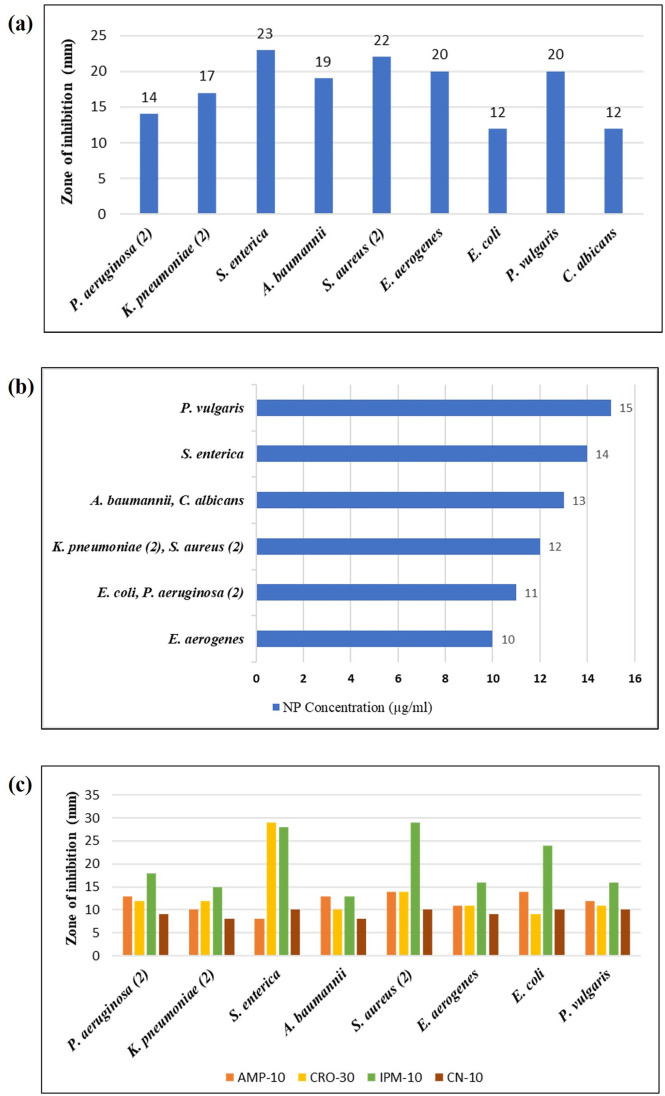
Graphs depicting (a) zones of inhibition of Fe_2_O_3_ nanoparticles against microbial isolates, (b) minimum inhibitory concentration of Fe_2_O_3_ nanoparticles (c) zones of inhibition for combined antibacterial potential of Fe_2_O_3_ nanoparticles and selected antibiotics.

Crude *P*. *emblica* fruit extract didn’t display considerable antimicrobial efficacy (Fig 5a). Conversely, *P*. *emblica*-mediated Fe_2_O_3_ nanoparticles exhibited impressive antimicrobial capability against Gram-positive as well as Gram-negative isolates of bacteria in addition to the fungal isolate (Fig 5b). The least inhibition zone (12 mm) was documented against *E*. *coli* and *C*. *albicans*, while the greatest inhibition zone (23 mm) was noticed against *S*. *enterica* (Fig 6a). According to the CLSI guidelines, these results indicate that the isolated microbes were susceptible to the synthesized nanoparticles. For all bacteria, one isolate was involved in this study except *P*. *aeruginosa*, *K*. *pneumoniae* and *S*. *aureus*, for which two isolates were considered and mean diameters for inhibition zones are included herein. MIC value for Fe_2_O_3_ nanoparticles was observed in the range of 10–15 μg/ml for various microbes. The least MIC value (10 μg/ml) was recorded with regard to *E*. *aerogenes*, while the highest MIC value (15 μg/ml) corresponded to *P*. *vulgaris* (Fig 6b).

### Antibiotic susceptibility testing

Several antibiotics were utilized for assessing the sensitivity of bacterial isolates, as shown in Table 1. Among these isolates, only *S*. *aureus* and *E*. *coli* exhibited sensitivity to 3 of the 9 antibiotics that were included. On the other hand, *P*. *aeruginosa*, *A*. *baumannii*, and *E*. *aerogenes* displayed sensitivity to just 1 of the selected antibiotics, whereas *K*. *pneumoniae*, *S*. *enterica*, and *P*. *vulgaris* were entirely resistant to the antibiotics included in investigation. Notably, ciprofloxacin proved ineffective against all isolates, while ceftriaxone exhibited effectiveness against one isolate of *S*. *aureus* only.

**Table 1:** Antibiotic sensitivity testing of isolated bacterial strains.

Isolate	Clindamycin (CL-10)	Imipenem (IPM-10)	Ciprofloxaci n (CIP-5)	Gentamicin (CN-10)	Ceftriaxone (CRO-30)	Cefepime (FEP-30)	Sulbactam (SCF-105)	Trimethoprim/Sulfamethoxazole (SXT-25)	Ampicillin (AMP-10)
*P*. *aeruginosa*-1	S	R	R	R	R	R	R	-	R
*P*. *aeruginosa*-2	S	R	R	R	R	R	-	-	R
*K*. *pneumoniae*-1	-	S	R	R	R	-	R	-	R
*K*. *pneumoniae*-2	-	R	R	R	R	R	-	R	R
*S*. *enterica*	IS	-	R	-	R	-	-	R	S
*A*. *baumannii*	IS	R	R	R	R	R	S	R	-
*S*. *aureus*-1	-	S	-	S	R	-	-	S	R
*S*. *aureus*-2	-	S	-	R	S	-	-	S	IS
*E*. *aerogenes*	S	R	R	R	R	R	R	-	IS
*E*. *coli*	-	S	R	S	R	S	-	R	R
*P*. *vulgaris*	-	R	R	R	R	R	-	R	R

### Synergistic activity of nanoparticles with antibiotics

Zones of inhibition regarding Fe_2_O_3_ nanoparticles combined with different antibiotics (AMP-10, CRO-30, IPM-10, CN-10) were in the range of 8-29mm (Fig 6c). Specifically, the combination of Fe_2_O_3_ nanoparticles with AMP-10 and CN-10 showed inhibition zones ranging from 8 to 14 mm, which means that most of the isolates were resistant or intermediate sensitive to these combinations. CRO-30 and IPM-10 with Fe_2_O_3_ nanoparticles exhibited good antibacterial activity with zones of inhibition ranging between 9 mm and 29 mm. *S*. *enterica* showed the highest sensitivity to Fe_2_O_3_ nanoparticles combined with CRO-30 and IPM-10, showing 29 mm and 28 mm zones of inhibition, respectively. *S*. *aureus* and *E*. *coli* showed high sensitivity to only one combination ie, IPM-10 and Fe_2_O_3_ nanoparticles as manifested by the zones of inhibition measuring 29 mm and 24 mm, respectively (Fig 5c). When these results were compared to the individual results of nanoparticles, and antibiotics, no synergistic effect was noticed. Results indicated that the antibacterial activity of Fe_2_O_3_ nanoparticles was marginally reduced when they were combined with the antibiotics, which implies no synergy between them. All of the calculated FICI values were above 1, which also reinforced these observations, indicating no synergy. Nanoparticles alone proved to be the most effective against all bacterial isolates included in the study.

### Photocatalytic dye degradation

**Fig 7:**
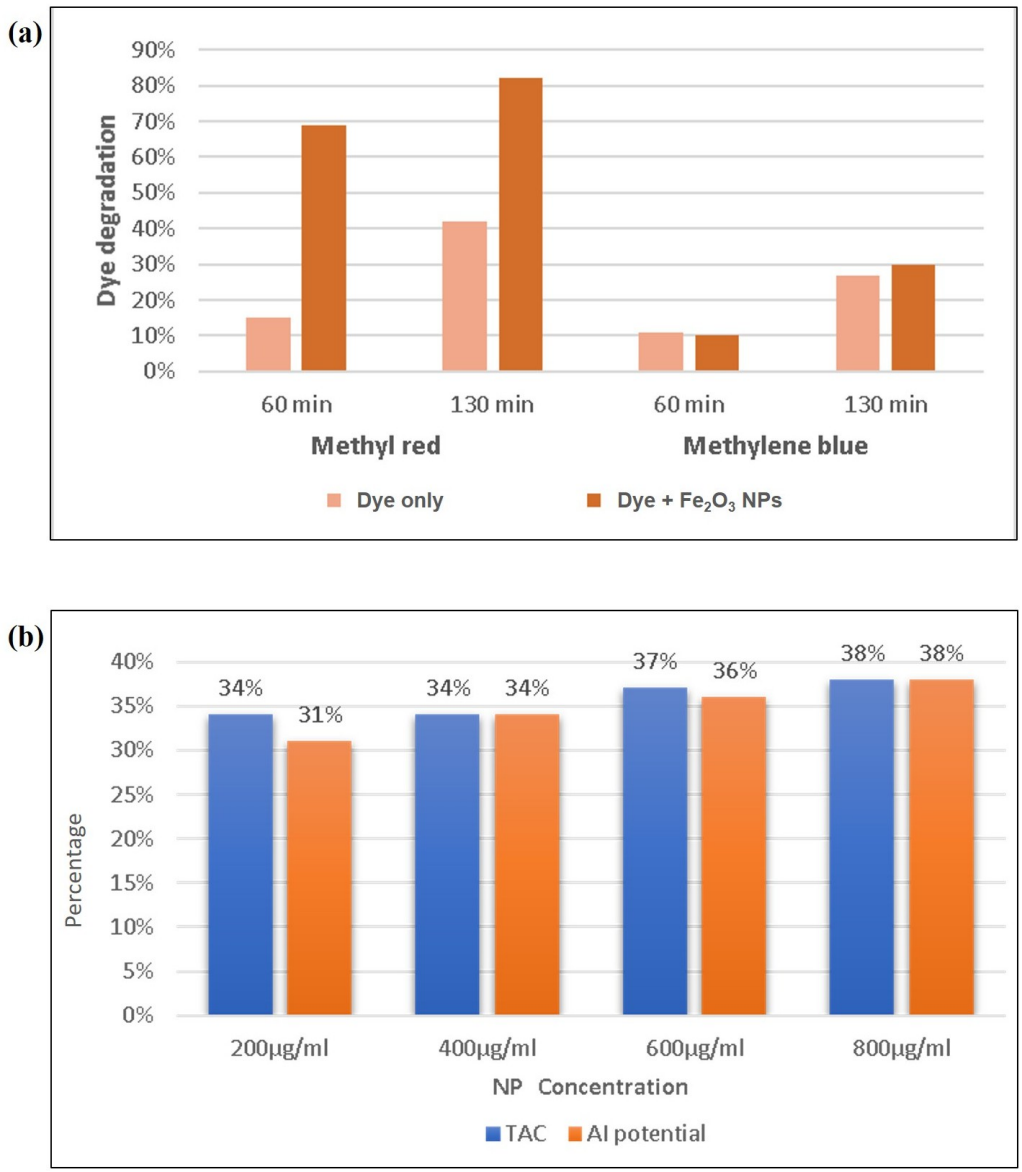
Graphs depicting (a) photocatalytic degradation of methyl red and methylene blue in the presence and absence of Fe_2_O_3_ nanoparticles, (b) total antioxidant capacity and anti-inflammatory potential of Fe_2_O_3_ nanoparticles.

Metal oxide nanoparticles can play a significant role in the environmental remediation by acting as photo-catalysts to degrade synthetic dyes. Therefore, we explored the dye degradation potential of synthesized nanoparticles. When sunlight was present, Fe_2_O_3_ nanoparticles served as effective catalysts for degrading methylene blue and methyl red dyes. Following a 60-minute exposure to sunlight, the degradation of methyl red reached 15% in the absence of nanoparticles, while it enormously increased to 69% when Fe_2_O_3_ nanoparticles were introduced (Fig 7a). After 130 minutes, the degradation of methyl red without nanoparticles reached 42%, while the presence of Fe_2_O_3_ nanoparticles resulted in an impressive 82% degradation of the dye under identical conditions. On the other hand, methylene blue degradation post 130 minutes, reached 27% in the absence of nanoparticles and showed a marginal increase to 30% when Fe_2_O_3_ nanoparticles were added.

### Total antioxidant capacity and anti-inflammatory activity

Fe_2_O_3_ nanoparticles manifested notable antioxidant activity, which was found to correlate with increasing nanoparticle concentration. This enhanced antioxidant activity is attributed to the polyphenolic constituents of *P*. *emblica* fruit extract used to synthesize nanoparticles. Specifically, at conc. of 200 μg/ml, 400 μg/ml, 600μg/ml, and 800 μg/ml, Fe_2_O_3_ nanoparticles demonstrated total antioxidant activity levels of 34%, 34%, 37%, and 38%, respectively (Fig 7b). Furthermore, Fe_2_O_3_ nanoparticles also demonstrated significant anti-inflammatory potential, with their effectiveness increasing in proportion to the nanoparticle conc. At conc. of 200 μg/ml, 400 μg/ml, 600μg/ml, and 800 μg/ml, Fe_2_O_3_ nanoparticles exhibited anti-inflammatory potential levels of 31%, 34%, 36%, and 38%, respectively (Fig 7b). Both the antioxidant and anti-inflammatory capabilities were determined in relation to ascorbic acid as standard reference. It is noteworthy that the absorbance values calculated for Fe_2_O_3_ nanoparticles in antioxidant and anti-inflammatory activities were inferior to those calculated for ascorbic acid at equivalent conc.

## Discussion

The biosynthesis of Fe_2_O_3_ nanoparticles started when aqueous extract of *P*. *emblica* fruits was mixed with the iron salt (ferric chloride) solution, leading to a rapid and distinct color change from yellowish brown to intense black. The phytochemicals present within the extract manifested a fundamental role in directing the synthesis of nanoparticles by causing their reduction and stabilization, as well as by impacting their morphology and properties [28].

A comparable change in color was noticed by Bhuiyan et al and Adhikari et al while synthesizing Fe_2_O_3_ nanoparticles from *Carica papaya* and *Psidium guajava* leaf extracts, respectively [13, 14]. Both of these studies utilized ferric chloride hexahydrate as a precursor for ferric ions for synthesizing the nanoparticles. Although the green source used for the production of Fe_2_O_3_ nanoparticles was different in the current and previous studies, the final color of the Fe_2_O_3_ nanoparticles solution was exactly the same. This change in color could be explained in terms of a phenomenon termed SPR that happens on the exterior face of metallic nanoparticles. It was established by UV-Vis spectroscopy which displayed sharp peak at about 295 nm, which was closely related to the one previously documented (around 298-301nm) by Karpagavinayagam and Vedhi who synthesized Fe_2_O_3_ nanoparticles using *Avicennia Marina* flower extract [29]. In another study, Da’na et al synthesized Fe_2_O_3_ nanoparticles using *Acacia nilotica* seedless pods and reported two absorption bands at around 217 nm and 283 nm, out of which one peak is in similar position to that reported in the current study [30].

FTIR spectrophotometry of *P*. *emblica*-mediated Fe_2_O_3_ nanoparticles exhibited peaks at 3165 cm^−1^ and 1610 cm^−1^ which pointed towards O-H bond and C = C bond, correspondingly. Peaks found in the range of 400–600 cm^−1^ specified the occurrence of Fe-O bond further validating the formation of Fe_2_O_3_ nanoparticles [31]. Amutha and Sridhar synthesized Fe_2_O_3_ nanoparticles from *Glycosmis mauritiana* leaf extract and reported similar FTIR results with peaks at 3334 cm^−1^, 2115 cm^−1^ as well as 1628 cm^−1^ directing towards—OH, C≡N and C = C groups, correspondingly [32]. These observations indicated that several bio-active phytomolecules may be found on nanoparticle surface, and these led to the development of several peaks at various wavenumbers. Adhikari et al also documented similar FTIR results regarding green synthesized Fe_2_O_3_ nanoparticles [13]. The elemental constitution of Fe_2_O_3_ nanoparticles was divulged through EDX, which presented two distinct K-α peaks before 0.8 keV attributing to Fe atoms and two more peaks between 0.0–0.7keV corresponding to C and O atoms. Fe and O were the most abundant elements present in the sample collectively constituting 54% of the total sample weight. Shahid et al also reported that Fe and O accounted for 51% of the total atomic mass of the Fe_2_O_3_ nanoparticles synthesized from honey [26]. The residues of ferric chloride and sodium hydroxide utilized during nanoparticle synthesis were also found in the sample. The presence of such residues has also been documented in a previous study [33].

XRD analysis was done to obtain information regarding the crystal structure of the nanoparticles. By comparing the strengths of the experimental and reference peaks in a diffraction pattern, the amount of iron oxide that has developed in a mixture can be determined [34]. Fe_2_O_3_ nanoparticles showed XRD peaks at 2 (θ) values of 24.1, 33.2, 35.7, 41.2 and 49.78. Similar XRD patterns of Fe_2_O_3_ nanoparticles synthesized from *G*. *mauritiana* aqueous extract have been reported by Amutha and Sridhar [32]. SEM images of the synthesized nanoparticles showed that they were not uniform in shape and formed large irregular aggregates. Comparable outcomes were reported by Adhikari et al and Jain et al [13, 35]. Agglomeration of Fe_2_O_3_ nanoparticles could be due to the molecular clustering of several bioactive reducing agents that were present in the plant extract. Another possible reason can be the tendency of Fe_2_O_3_ nanoparticles to agglomerate due to their magnetic interactions [36].

Significant antimicrobial activity of Fe_2_O_3_ nanoparticles produced from distinct organic extracts was described formerly against different bacteria like *S*. *aureus*, *E*. *coli*, *B*. *cereus*, and *Pseudomonas* spp [37]. *P*. *emblica*-mediated Fe_2_O_3_ nanoparticles synthesized in the present study also demonstrated good antimicrobial potential with zones of inhibition ranging from 12 mm to 23 mm against various clinically isolated microbes. MIC value for Fe_2_O_3_ nanoparticles was observed in the range of 10–15 μg/ml. In a previous study, Pallela et al synthesized Fe_2_O_3_ nanoparticles using *Sida cordifolia* extract and reported zones of inhibition measuring 16 mm, 13 mm, 11mm and 12 mm against *B*. *subtilis*, *S*. *aureus*, *E*. *coli*, and *K*. *pneumoniae*, correspondingly [38]. Veeramanikandan et al also reported similar observations [39]. In a previous study, Das et al investigated the antibacterial activity of Fe_2_O_3_ nanoparticles synthesized through thermal decomposition of a diiron (III) complex precursor and reported the zones of inhibition against *S*. *aureus*, *P*. *vulgaris* and *P*. *aeruginosa* as 25 mm, 12 mm and 17 mm, respectively, while MIC value against *S*. *aureus* was determined as 12.5 μg/ml [40]. In current study, under similar conditions, the zones of inhibition measured against *S*. *aureus*, *P*. *vulgaris* and *P*. *aeruginosa* were 22 mm, 20 mm and 14 mm, respectively, while the MIC value against *S*. *aureus* was 12 μg/ml. Khatami et al documented MIC value of Fe_2_O_3_ nanoparticles synthesized with *Rosmarinus officinalis* leaves, against *E*. *coli*, as 250 μg/ml while the present study reported MIC against *E*. *coli* as 11 μg/ml [41]. Fe_2_O_3_ nanoparticles synthesized by Adhikari et al showed similar anti-fungal zones of inhibition for *C*. *albicans* (13 mm) as those reported in the present study (12 mm) [13]. A comparison of these results suggests that there could be a discrepancy in the outcomes attributing to the variations in the composition of green source, the protocols used for synthesis and the approach adopted for nanoparticle production. In addition, the nature of test microbes and the nanoparticle concentration applied for testing are equally important.

Antibiotic sensitivity testing of the chosen bacterial strains indicated that they possessed resistance against several drugs, classifying them as "multi-drug resistant bacteria". Similar findings were described by Wang et al who observed the occurrence of MDR pathogens in patients suffering from hospital-acquired infections [42]. These bacteria were resistant to antibiotics like ampicillin, ceftriaxone, ciprofloxacin, and imipenem but were sensitive to amikacin and gentamicin. This pattern closely aligns with the observations of the present study. To deal with the antibiotic resistance problem, some new approaches have been tested. One of these is the fusion of metallic nanoparticles with antimicrobial substances and it has shown great promise. By employing nanotechnology, the interaction between nanoparticles and bacteria can be harnessed to enhance the efficacy of microbial eradication. This cutting-edge method combines metal oxide nanoparticles with antimicrobial agents, creating a new class of drugs that are more effective against drug-resistant bacteria [43]. By using the same concept, *P*. *emblica*-mediated Fe_2_O_3_ nanoparticles were used in combination with antibiotics for exploration of their antibacterial potential. Unfortunately, the nanoparticles didn’t manifest synergy with any of the tested antibiotics as uncovered by FICI values. Shahid et al described similar results regarding honey mediated Fe_2_O_3_ nanoparticles [26]. In contrast, research by Nishanthi et al highlighted effective synergy when gold, silver, and platinum nanoparticles were combined with various antibacterials [44]. This increased effectiveness could stem from the formation of bonds between the antibiotics and nanoparticles.

Our synthesized Fe_2_O_3_ nanoparticles enhanced the degradation of selected dyes in the presence of sunlight. In a well-defined time period, methyl red degradation was 42% without nanoparticles which increased to 82% in the presence of Fe_2_O_3_ nanoparticles. Methylene blue degradation was 27% without nanoparticles which barely increased to 30% in the presence of Fe_2_O_3_ nanoparticles. In a previous report, Bishnoi et al researched the photocatalytic degradation of methylene blue dye using *Cynometra ramiflora*-derived Fe_2_O_3_ nanoparticles as a catalyst and calculated the photocatalytic rate constant as 0.0253 min^–1^ [45]. In the current study, Fe_2_O_3_ nanoparticles showed comparatively less degradation of methylene blue with photocatalytic rate constant calculated as 0.0110 min^–1^. For methyl red dye, the photocatalytic rate constant was 0.0436 min^–1^ which meant that Fe_2_O_3_ nanoparticles were more effective in the degradation of methyl red dye as compared to methylene blue dye. In another study, *Catharanthus roseus*-mediated Fe_2_O_3_ nanoparticles were tested for photocatalytic degradation of methyl orange dye. While noting results at uniform time intervals for 2 hours, a maximum of 50% dye degradation was observed [46]. It proves the competency of green-synthesized Fe_2_O_3_ nanoparticles in environmental dye degradation. Moreover, it was inferred that dye degradation declines with time which may be due to the binding of degraded material to the nanoparticle surface.

*P*. *emblica*-mediated Fe_2_O_3_ nanoparticles exhibited notable antioxidant and anti-inflammatory activities, which were found to correlate with the increasing nanoparticle concentration. These activities are ascribed to the phenolic constituents present in the *P*. *emblica* fruit extract. Both the total antioxidant capacity and anti-inflammatory activity were determined utilizing ascorbic acid as standard and were in the range of 30–40%. The absorbance values calculated for Fe_2_O_3_ nanoparticles were lower in all cases than those calculated for ascorbic acid at equivalent conc. In previous research, significant antioxidant potential of superparamagnetic Fe_2_O_3_ nanoparticles synthesized from aqueous extract of tea-pruning waste and *Stevia* plant has been reported [47, 48]. A contemporary study documented good anti-inflammatory potential of biosynthesized iron oxide and zinc oxide nano-ointment [49]. A variation in results may appear due to the use of differing sources of plant and techniques for nanoparticles synthesis. Hence, *P*. *emblica*-mediated Fe_2_O_3_ nanoparticles have been found to possess comparable, and sometimes even, superior qualities to those synthesized by conventional methods, opening new avenues for their applications in various fields.

The scope of present investigation was restricted to the exploration of biological effects of synthesized nanoparticles in a controlled lab setting, without delving into the specifics of how they function. Insight into the precise mechanisms through which these nanoparticles operate would enhance our comprehension of their characteristics and potential uses. Additionally, refining the nanoparticle synthesis process is essential to achieving a consistent batch of nanoparticles with minimal size variability. It is imperative for subsequent studies to focus on the biosafety and biocompatibility of these nanoparticles to verify their purity and ensure their non-toxic nature.

## Conclusion

The present research represents a simple, reproducible and environment friendly method for producing Fe_2_O_3_ nanoparticles, utilizing the fruit extract of *P*. *emblica* as reducing and stabilizing agent. Synthesized nanoparticles underwent a thorough characterization process through various analytical methods, including UV-Vis spectroscopy, FTIR, XRD, EDX, and SEM. Remarkably, the nanoparticles exhibited superior antimicrobial effects against diverse clinical pathogens when compared to several standard antibiotics. Furthermore, the *P*. *emblica*-derived Fe_2_O_3_ nanoparticles demonstrated promising antioxidant and anti-inflammatory properties. They enhanced the photocatalytic degradation of methylene blue and methyl red dyes, indicating their potential application in both medicine and environment. Hence, the green synthesis of *P*. *emblica* fruit extract mediated Fe_2_O_3_ nanoparticles is a testament to the innovative approaches within nanotechnology aimed at harmonizing technological advancement with environmental stewardship. As research in this area progresses, it holds the promise for revolutionizing the production and application of nanoparticles, with profound implications for both science and society.

## Supporting information

S1 Graphical abstract (TIF)

## References

[1] Nasrollahzadeh M, Sajadi SM, Sajjadi M, Issaabadi Z. An introduction to nanotechnology. In: Interface science and technology. Elsevier; 2019. pp. 1–27.

[2] McNeil SE. Nanotechnology for the biologist. J Leukoc Biol. 2005. doi: 10.1189/jlb.0205074 15923216

[3] Khan I, Saeed K, Khan I. Nanoparticles: properties, applications and toxicities. Arab J Chem. 2019. doi: 10.1016/j.arabjc.2017.05.011

[4] Sim S, Wong NK. Nanotechnology and its use in imaging and drug delivery (Review). Biomed Rep. 2021. doi: 10.3892/br.2021.1418 33728048 PMC7953199

[5] Saif S, Tahir A, Chen Y. Green synthesis of iron nanoparticles and their environmental applications and implications. Nanomaterials (Basel). 2016. doi: 10.3390/nano6110209 28335338 PMC5245755

[6] Siddiqi KS, Ur Rahman A, Tajuddin, Husen A. Biogenic fabrication of iron/iron oxide nanoparticles and their application. Nanoscale Res Lett. 2016. doi: 10.1186/s11671-016-1714-0 27837567 PMC5106417

[7] Ali A, Zafar H, Zia M, Haq I, Phull AR, Ali JS, et al. Synthesis, characterization, applications, and challenges of iron oxide nanoparticles. Nanotechnol Sci Appl. 2016. doi: 10.2147/NSA.S99986 27578966 PMC4998023

[8] Wu W, Wu Z, Yu T, Jiang C, Kim WS. Recent progress on magnetic iron oxide nanoparticles: synthesis, surface functional strategies and biomedical applications. Sci Technol Adv Mater. 2015. doi: 10.1088/1468-6996/16/2/023501 27877761 PMC5036481

[9] Jamkhande PG, Ghule NW, Bamer AH, Kalaskar MG. Metal nanoparticles synthesis: An overview on methods of preparation, advantages and disadvantages, and applications. J Drug Deliv Technol. 2019. doi: 10.1016/j.jddst.2019.101174

[10] Hussain I, Singh NB, Singh A, Singh H, Singh SC. Green synthesis of nanoparticles and its potential application. Biotechnol Lett. 2016. doi: 10.1007/s10529-015-2026-7 26721237

[11] Singh J, Kaur G, Kaur P, Bajaj R, Rawat M. A review on green synthesis and characterization of silver nanoparticles and their applications: a green nanoworld. World J Pharm Pharm Sci. 2016. (10.20959/wjpps20167-7227).

[12] Thakkar KN, Mhatre SS, Parikh RY. Biological synthesis of metallic nanoparticles. Nanomedicine. 2010. doi: 10.1016/j.nano.2009.07.002 19616126

[13] Adhikari A, Chhetri K, Acharya D, Pant B, Adhikari A. Green synthesis of iron oxide nanoparticles using *Psidium guajava* L. leaves extract for degradation of organic dyes and anti-microbial applications. Catalysts. 2022. doi: 10.3390/catal12101188

[14] Bhuiyan MSH, Miah MY, Paul SC, Aka TD, Saha O, Rahaman M, et al. Green synthesis of iron oxide nanoparticle using *Carica papaya* leaf extract: application for photocatalytic degradation of remazol yellow RR dye and antibacterial activity. Heliyon. 2020. doi: 10.1016/j.heliyon.2020.e04603 32775754 PMC7404534

[15] Jamzad M, Bidkorpeh MK. Green synthesis of iron oxide nanoparticles by the aqueous extract of *Laurus nobilis* L. leaves and evaluation of the antimicrobial activity. J Nanostructure Chem. 2020. doi: 10.1007/s40097-020-00341-1

[16] Caroling G, Vinodhini E, Ranjitham AM, Shanthi P. Biosynthesis of copper nanoparticles using aqueous *Phyllanthus embilica* (Gooseberry) extract-characterisation and study of antimicrobial effects. Int J Nano Chem. 2015;1(2): 53–63.

[17] Yadav SS, Singh MK, Singh PK, Kumar V. Traditional knowledge to clinical trials: A review on therapeutic actions of *Emblica officinalis*. Biomed Pharmacother. 2017. doi: 10.1016/j.biopha.2017.07.065 28747010

[18] Ananda A, Ramakrishnappa T, Archana S, Reddy Yadav LS, Shilpa BM, Nagaraju G, et al. Green synthesis of MgO nanoparticles using *Phyllanthus emblica* for Evans blue degradation and antibacterial activity. Mater Today: Proc. 2022. doi: 10.1016/j.matpr.2021.05.340

[19] Renuka R, Devi KR, Sivakami M, Thilagavathi T, Uthrakumar R, Kaviyarasu K. Biosynthesis of silver nanoparticles using *Phyllanthus emblica* fruit extract for antimicrobial application. Biocatal Agri Biotechnol. 2020. doi: 10.1016/j.bcab.2020.101567

[20] Sathishkumar M, Saroja M, Venkatachalam M, Rajamanickam A. Biosynthesis of zinc sulphide nanoparticles using *Phyllanthus emblica* and their antimicrobial activities. Elixir Elec Eng. 2017;102: 44411–44415.

[21] Thoidingjam S, Tiku AB. Therapeutic efficacy of *Phyllanthus emblica*-coated iron oxide nanoparticles in A549 lung cancer cell line. Nanomedicine. 2019;14(17): 2355–2371. 31414606 10.2217/nnm-2019-0111

[22] Wang R, Xu X, Puja AM, Perumalsamy H, Balusamy SR, Kim H, et al. Gold nanoparticles prepared with *Phyllanthus emblica* fruit extract and *Bifidobacterium animalis* subsp. Lactis can induce apoptosis via mitochondrial impairment with inhibition of autophagy in the human gastric carcinoma cell line AGS. Nanomaterials (Basel). 2021. doi: 10.3390/nano11051260 34064899 PMC8150816

[23] Ovais M, Khalil AT, Islam NU, Ayaz M, Sravanan M, Ahmad I, et al. Role of plant phytochemicals and microbial enzymes in biosynthesis of metallic nanoparticles. Appl Microbiol Biotechnol. 2018. doi: 10.1007/s00253-018-9146-7 29882162

[24] Ying S, Guan Z, Ofoegbu PC, Clubb P, Rico C, He F, et al. Green synthesis of nanoparticles: current developments and limitations. Environ Technol Innov. 2022. doi: 10.1016/j.eti.2022.102336

[25] Iravani S. Green synthesis of metal nanoparticles using plants. Green Chem. 2011. doi: 10.1039/C1GC15386B

[26] Shahid H, Shah AA, Shah Bukhari SNU, Naqvi AZ, Arooj I, Javeed M, et al. Synthesis, characterization, and biological properties of iron oxide nanoparticles synthesized from *Apis mellifera* honey. Molecules. 2023. doi: 10.3390/molecules28186504 37764280 PMC10534332

[27] Dulta K, Ağçeli KG, Chauhan P, Jasrotia R, Chauhan PK, Ighalo JO. Multifunctional CuO nanoparticles with enhanced photocatalytic dye degradation and antibacterial activity. Sustain Environ Res. 2022. doi: 10.1186/s42834-021-00111-w

[28] Soni V, Raizada P, Singh P, Cuong HN, Rangabhashiyam S, Saini A, et al. Sustainable and green trends in using plant extracts for the synthesis of biogenic metal nanoparticles toward environmental and pharmaceutical advances: A review. Environ Res. 2021;202: 111622. doi: 10.1016/j.envres.2021.111622 34245729

[29] Karpagavinayagam P, Vedhi C. Green synthesis of iron oxide nanoparticles using *Avicennia marina* flower extract. Vacuum. 2019. doi: 10.1016/j.vacuum.2018.11.043

[30] Da’na E, Taha A, Afkar E. Green synthesis of iron nanoparticles by *Acacia nilotica* pods extract and its catalytic, adsorption, and antibacterial activities. Appl Sci. 2018. doi: 10.3390/app8101922

[31] Zambri NDS, Taib NI, Abdul Latif F, Mohamed Z. Utilization of neem leaf extract on biosynthesis of iron oxide nanoparticles. Molecules. 2019. doi: 10.3390/molecules24203803 31652583 PMC6832892

[32] Amutha S, Sridhar S. Green synthesis of magnetic iron oxide nanoparticle using leaves of *Glycosmis mauritiana* and their antibacterial activity against human pathogens. J Innov Pharm Biol Sci. 2018;5(2): 22–26.

[33] Qasim S, Zafar A, Saif MS, Ali Z, Nazar M, Waqas M, et al. Green synthesis of iron oxide nanorods using *Withania coagulans* extract improved photocatalytic degradation and antimicrobial activity. J Photochem Photobiol B. 2020. doi: 10.1016/j.jphotobiol.2020.111784 31954266

[34] Laurent S, Forge D, Port M, Roch A, Robic C, Elst LV, et al. Magnetic iron oxide nanoparticles: synthesis, stabilization, vectorization, physicochemical characterizations, and biological applications. Chem Rev. 2008;108(6): 2064–2110. doi: 10.1021/cr068445e 18543879

[35] Jain R, Mendiratta S, Kumar L, Srivastava A. Green synthesis of iron nanoparticles using *Artocarpus heterophyllus* peel extract and their application as a heterogeneous Fenton-like catalyst for the degradation of Fuchsin Basic dye. Curr Res Green Sustain Chem. 2021. doi: 10.1016/j.crgsc.2021.100086

[36] Mørup S, Hansen MF, Frandsen C. Magnetic interactions between nanoparticles. Beilstein J Nanotechnol. 2010;1(1): 182–190. doi: 10.3762/bjnano.1.22 21977409 PMC3045912

[37] Kirdat PN, Dandge PB, Hagwane RM, Nikam AS, Mahadik SP, Jirange ST. Synthesis and characterization of ginger *(Z*. *officinale*) extract mediated iron oxide nanoparticles and its antibacterial activity. Mater Today: Proc. 2021. doi: 10.1016/j.matpr.2020.11.422

[38] Pallela P, Ummey S, Ruddaraju LK, Gadi S, Cherukuri CSL, Barla S, et al. Antibacterial efficacy of green synthesized alpha-Fe(2)O(3) nanoparticles using *Sida cordifolia* plant extract. Heliyon. 2019. doi: 10.1016/j.heliyon.2019.e02765 31799458 PMC6881625

[39] Veeramanikandan V, Madhu G, Pavithra V, Jaianand K, Balaji P. Green synthesis, characterization of iron oxide nanoparticles using *Leucas aspera* leaf extract and evaluation of antibacterial and antioxidant studies. Int J Agric Innov Res. 2017;6(2): 242–250.

[40] Das S, Diyali S, Vinothini G, Perumalsamy B, Balakrishnan G, Ramasamy T, et al. Synthesis, morphological analysis, antibacterial activity of iron oxide nanoparticles and the cytotoxic effect on lung cancer cell line. Heliyon. 2020. doi: 10.1016/j.heliyon.2020.e04953 33005785 PMC7511749

[41] Khatami M, Aflatoonian MR, Azizi H, Mosazade F, Hooshmand A, Nobre MAL, et al. Evaluation of antibacterial activity of iron oxide nanoparticles against *Escherichia coli*. Int J Basic Sci Med. 2017. doi: 10.15171/ijbsm.2017.31

[42] Wang M, Wei H, Zhao Y, Shang L, Di L, Lyu C, et al. Analysis of multidrug-resistant bacteria in 3223 patients with hospital-acquired infections (HAI) from a tertiary general hospital in China. Bosn J Basic Med Sci. 2019. doi: 10.17305/bjbms.2018.3826 30579325 PMC6387671

[43] Alavi M, Hamblin MR, Martinez F, Kennedy JF, Khan H. Synergistic combinations of metal, metal oxide, or metalloid nanoparticles plus antibiotics against resistant and non-resistant bacteria. Micro Nano Bio Aspects. 2022;1(1): 1–9.

[44] Nishanthi R, Malathi S, Palani P. Green synthesis and characterization of bioinspired silver, gold and platinum nanoparticles and evaluation of their synergistic antibacterial activity after combining with different classes of antibiotics. Mater Sci Eng C Mater Biol Appl. 2019. doi: 10.1016/j.msec.2018.11.050 30606583

[45] Bishnoi S, Kumar A, Selvaraj R. Facile synthesis of magnetic iron oxide nanoparticles using inedible *Cynometra ramiflora* fruit extract waste and their photocatalytic degradation of methylene blue dye. Mater Res Bull. 2018. doi: 10.1016/j.materresbull.2017.08.040

[46] Roy A, Singh V, Sharma S, Ali D, Azad AK, Kumar G, et al. Antibacterial and dye degradation activity of green synthesized iron nanoparticles. J Nanomater. 2022. doi: 10.1155/2022/3636481

[47] Periakaruppan R, Chen X, Thangaraj K, Jeyaraj A, Nguyen HH, Yu Y, et al. Utilization of tea resources with the production of superparamagnetic biogenic iron oxide nanoparticles and an assessment of their antioxidant activities. J Clean Prod. 2021. doi: 10.1016/j.jclepro.2020.123962

[48] Khatami M, Alijani HQ, Fakheri B, Mobasseri MM, Heydarpour M, Farahani ZK, et al. Super-paramagnetic iron oxide nanoparticles (SPIONs): Greener synthesis using Stevia plant and evaluation of its antioxidant properties. J Clean Prod. 2019. doi: 10.1016/j.jclepro.2018.10.182

[49] Yadav E, Yadav P, Verma A. Amelioration of full thickness dermal wounds by topical application of biofabricated zinc oxide and iron oxide nano-ointment in albino Winstar rats. J Drug Deliv Technol. 2021. doi: 10.1016/j.jddst.2021.102833

