# Supplementary figures and images for: Characterization and applications of iron oxide nanoparticles synthesized from Phyllanthus emblica fruit extract
Source: PLoS One. 2024 Sep 19;19(9):e0310728. doi: 10.1371/journal.pone.0310728 (PMC11412514; doi:10.1371/journal.pone.0310728)

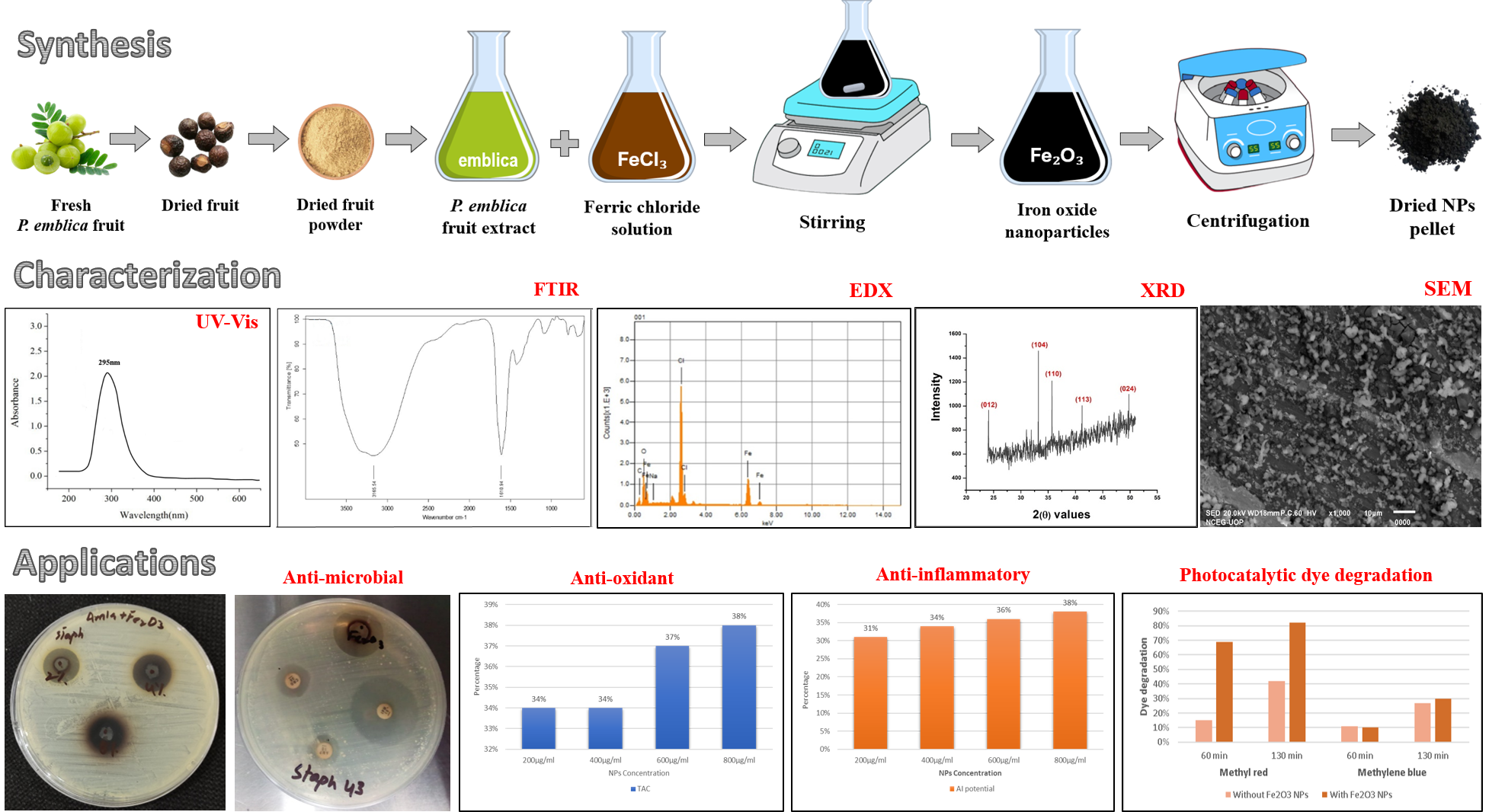

Supplement: S1 Graphical abstract — (TIF) [file pone.0310728.s001.tif]
